# Low plasma levels of miR-101 are associated with tumor progression in gastric cancer

**DOI:** 10.18632/oncotarget.20860

**Published:** 2017-09-13

**Authors:** Taisuke Imamura, Shuhei Komatsu, Daisuke Ichikawa, Mahito Miyamae, Wataru Okajima, Takuma Ohashi, Jun Kiuchi, Keiji Nishibeppu, Toshiyuki Kosuga, Hirotaka Konishi, Atsushi Shiozaki, Kazuma Okamoto, Hitoshi Fujiwara, Eigo Otsuji

**Affiliations:** ^1^ Division of Digestive Surgery, Department of Surgery, Kyoto Prefectural University of Medicine, Kawaramachihirokoji, Kamigyo-ku, Kyoto, 602-8566, Japan

**Keywords:** gastric cancer, tumor suppressor microRNA, plasma, biomarker

## Abstract

**Background:**

Several studies have identified the decreased expression of the tumor suppressor miR-101 in various cancers. In this study, we tested miR-101 as a potential therapeutic target and novel plasma biomarker for gastric cancer (GC).

**Results:**

The miR-101 expression level was significantly lower in GC tissues (*P* = 0.0038) and GC cell lines (*P* = 0.0238) than in normal gastric mucosa. Both exosomal and plasma miR-101 were significantly downregulated in GC patients compared with healthy volunteers (*P* = 0.0281 and *P* < 0.0001, respectively). Low miR-101 plasma level was significantly associated with advanced T factor, advanced disease stage, and peritoneal metastasis and predicted poor prognosis in GC patients (*P =* 0.0368; hazard ratio, 3.079; 95% confidence interval: 1.06–11.08). Overexpression of miR-101 in GC cells induced apoptosis by inhibiting MCL1 and suppressed cell migration and invasion by regulating ZEB1.

**Conclusions:**

Depletion of the tumor suppressor miRNA-101 in plasma is related to tumor progression and poor outcomes. Low plasma miR-101 may be a biomarker for GC, and its restoration might be a novel anticancer treatment strategy.

## INTRODUCTION

Gastric cancer (GC) is third leading cause of cancer-related death both globally and in Japan [1]. Although recent improvements in diagnostic techniques and perioperative management have increased early detection and decreased mortality over the past decades, GC remains one of the most common cancer types and constitutes a global health problem [1]. Advanced-stage GC patients in particular still demonstrate extremely poor survival rates [2]. Nevertheless, in clinical settings, no molecule has been used as an early diagnostic biomarker for GC, and only a few molecules have been validated as therapeutic targets [3–7]. Therefore, an understanding of the molecular mechanisms of tumorigenesis and the identification of both clinical biomarkers and molecular targets for GC are urgently needed to improve the survival rate of GC patients.

MicroRNAs (miRNAs), which are small noncoding RNAs, regulate the translation of specific protein-coding genes. Since their discovery in 1993 [8], numerous studies have demonstrated that alterations in miRNA expression correlate with the progression of various diseases, including several cancer types [9–12]. In recent decades, several studies have elucidated in detail the biological processes through which miRNAs become detectable in plasma/serum and remain in a remarkably stable form [10, 13–16]. Plasma/serum miRNAs become resistant to endogenous ribonuclease activity by binding to specific plasma proteins [17, 18] or being packaged into various types of secretory vesicles, including apoptotic bodies and exosomes in plasma/serum [13, 19–21]. Furthermore, multiple extracellular miRNAs have been shown to occur not only through cell lysis but also through active secretion [22–24], and these miRNAs can function as intercellular transmitters [16, 23, 25, 26]. Thus, various blood-based miRNAs have been identified as useful biomarkers for cancer patients [27–40].

Recently, Kosaka *et al.* suggested that some tumor suppressor miRNAs are secreted by healthy cells to prevent aberrant cell growth [41]. In our previous work, we identified that some tumor suppressor miRNAs in plasma, such as let-7a [27] and miR-375 [29, 30], were significantly downregulated in cancer patients compared with healthy volunteers. Because circulating miRNAs are thought to be released from both cancer tissues and normal tissues, most of these tumor suppressor miRNAs were thought to have been derived from normal tissues; thus, we hypothesized that tumor suppressor miRNAs become depleted from healthy cells according to cancer progression. Indeed, one of our previous studies showed that decreased plasma level of the tumor suppressor miR-375 in esophageal cancer patients was associated with worse survival [29]. Consequently, we proposed the novel theory that the downregulation of tumor suppressor miRNAs in the blood stream is correlated with tumor progression and poor prognostic outcomes.

In this study, we focused on the tumor suppressor miRNA-101, which targets multiple oncogenes and has been reported to be downregulated in various cancers. We clearly demonstrated that miRNA-101 plasma levels were depleted in GC patients and that this depletion was related to tumor progression and poor outcomes. Therefore, miRNA-101 plasma levels can be a useful biomarker for GC patients. Furthermore, the restoration of miR-101 in GC cells significantly inhibited tumor progression. Our results indicate that the systemic restoration and maintenance of the tumor suppressor miR-101 via nucleic acid medicine could be a novel therapeutic strategy for GC patients.

## RESULTS

### Study design to find depleted tumor suppressor miRNAs in GC patient plasma

**Figure 1:**
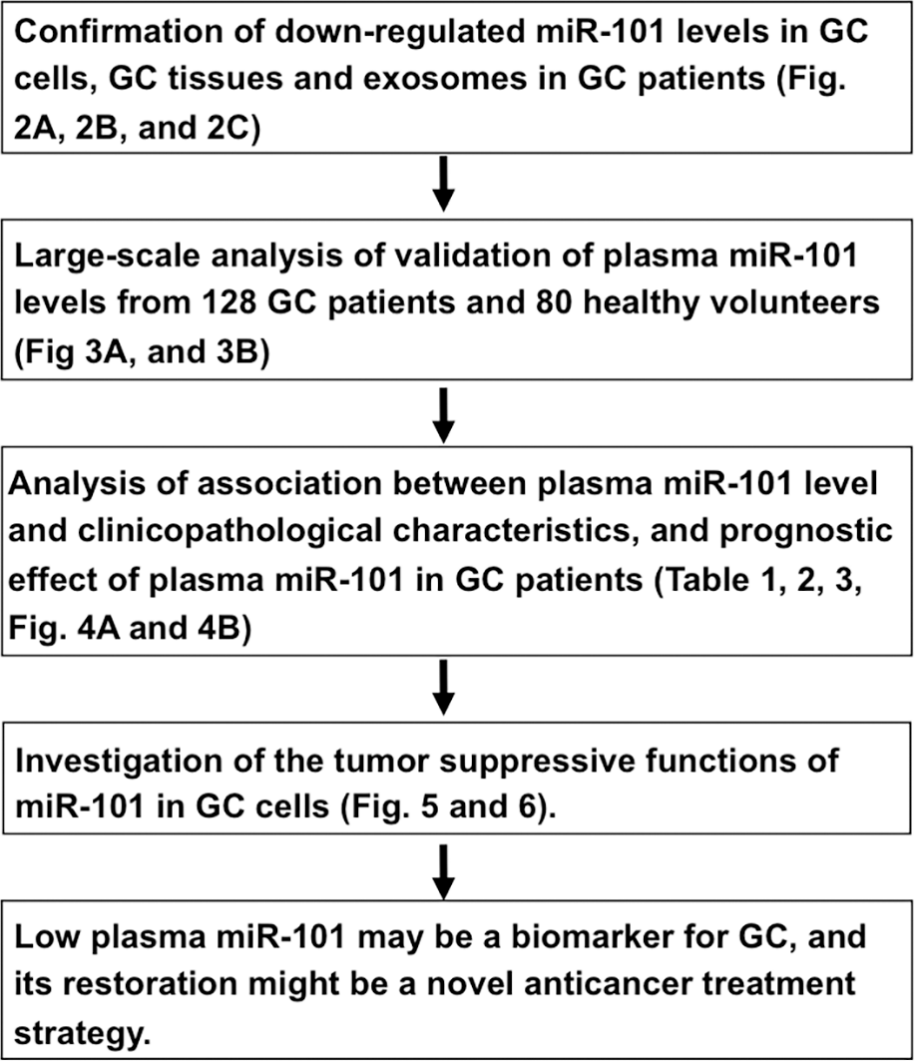
Study design to find depleted tumor suppressor miRNAs in GC patient plasma

As shown in Figure 1, this study was designed as follows: (1) evaluation of whether the plasma level of miR-101 reflects tumor dynamics and investigation of miR-101 expression in exosomes; (2) large-scale analysis to assess the miR-101 plasma level and investigate its association with clinicopathological characteristics and prognostic outcomes in GC patients; (3) evaluation of whether miR-101 overexpression in GC cells induces antitumor effects *in vitro*.

### Investigation into whether miR-101 plasma levels reflect tumor dynamics in plasma, gastric tissues, and exosomes

**Figure 2:**
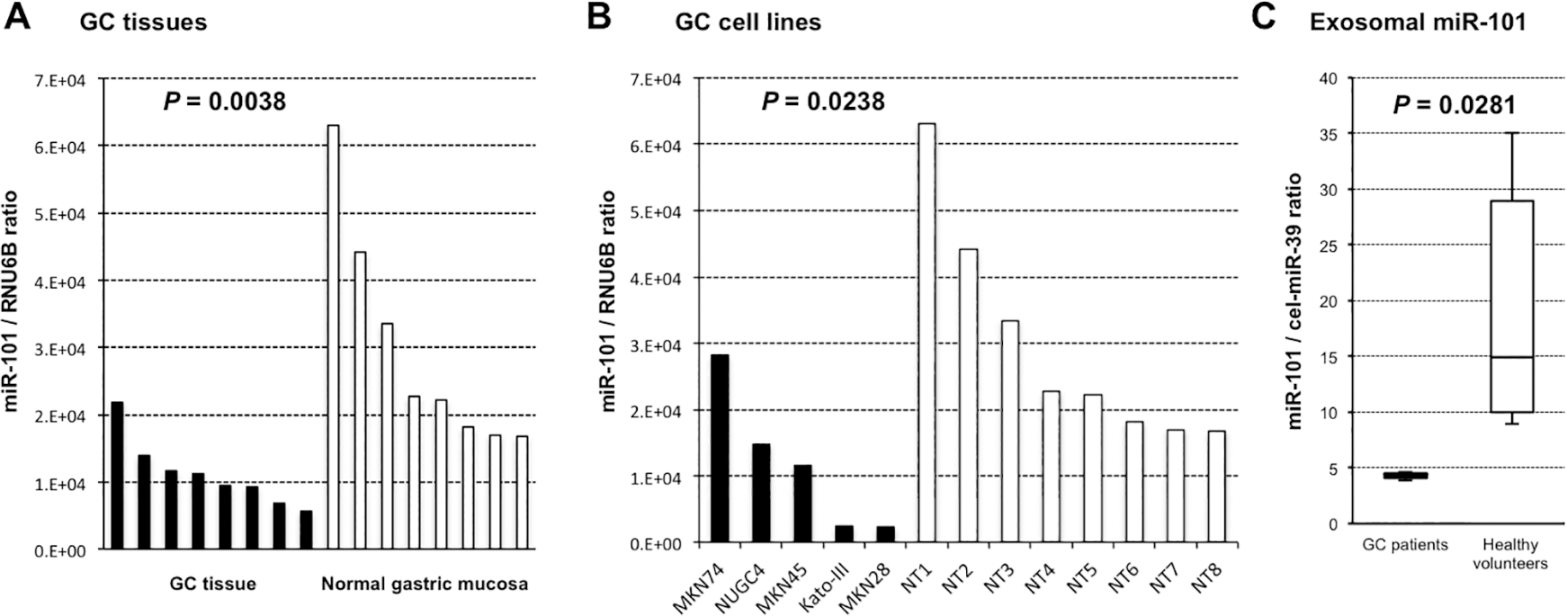
Expression level of miR-101 in GC tissues, GC cell lines, and exosomes of GC patients miR-101 expression was significantly lower in GC tissues (*P* = 0.0038) and GC cell lines (*P* = 0.0238) than in normal tissues (**A**, **B**). The exosomal miR-101 level was significantly lower in GC patients than in healthy volunteers (**C**).

To gain insight into whether miR-101 levels reflect tumor dynamics in gastric tissue and exosomes, we examined the expression levels of miR-101 in primary GC tissues and GC cell lines. We used qRT-PCR to determine the expression of miR-101 in eight GC tissues and eight normal gastric mucosa, as well as in the human GC cell lines Kato-III, NUGC4, MKN28, MKN45, and MKN74. The miR-101 expression level was significantly lower in GC tissues than in normal gastric mucosa (*P* = 0.0038) (Figure 2A). A similar result was observed in the GC cell lines in comparison with the normal gastric tissues (*P* = 0.0238) (Figure 2B).

Finally, we compared the miR-101 expression levels in exosomes extracted from the plasma of four consecutive GC patients and four healthy volunteers. As shown in Figure 2C, the level of exosomal miR-101 was significantly downregulated in GC patients compared with that in healthy volunteers (*P* = 0.0281). These results indicated that miR-101 levels reflect tumor dynamics and that miR-101 may be incorporated into exosomes and released into the plasma.

### Large-scale analysis of miR-101 plasma level in GC patients

**Figure 3:**
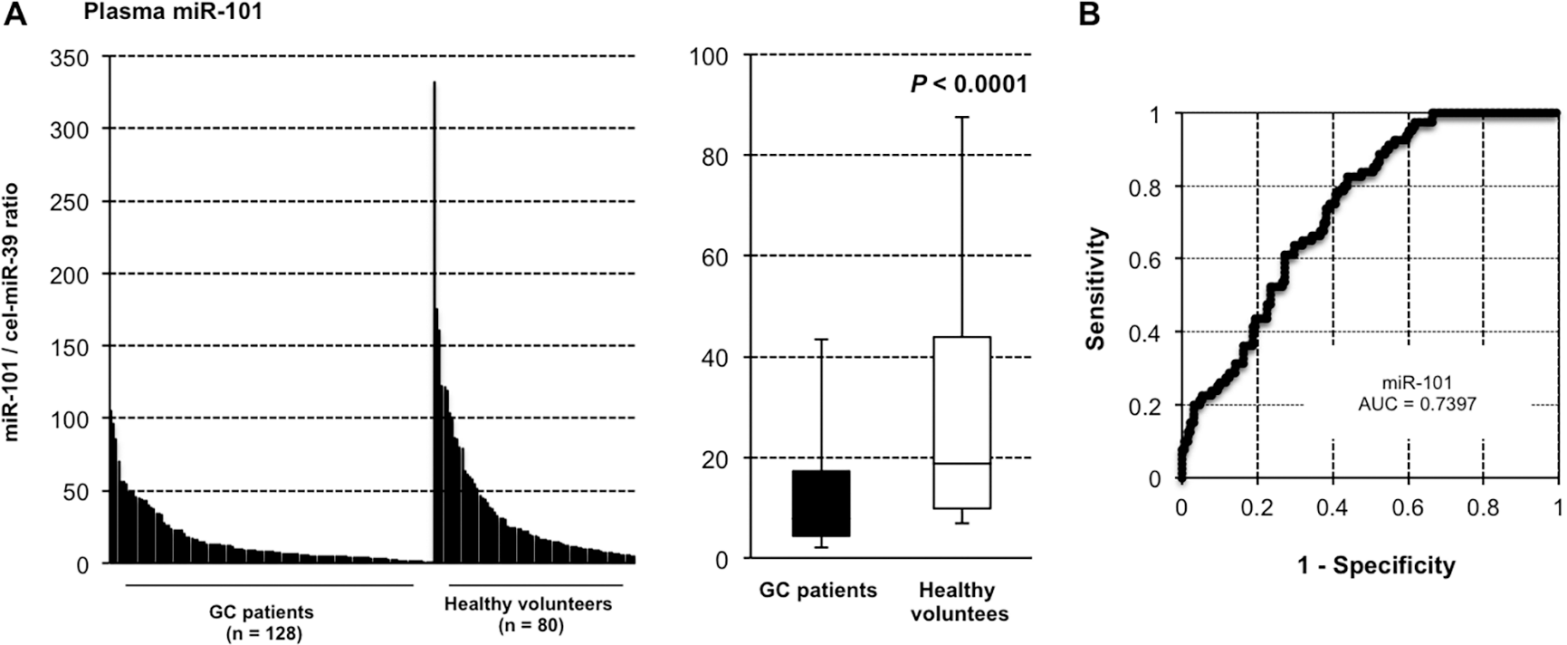
miR-101 was less expressed in the plasma of GC patients than in that of healthy volunteers For large-scale analysis, total RNA extracted from plasma samples of 128 GC patients and 80 age-matched healthy volunteers was used to analyze the expression level of miR-101 using qRT-PCR (**A**). Analysis of receiver-operating characteristic (ROC) curves was used to detect GC patients. ROC analysis showed a maximum AUC of 0.7397 for miR-101 (**B**).

We assessed miR-101 plasma level in a large-scale setting. Plasma miR-101 was detectable in all samples from 128 GC patients and 80 healthy volunteers. We observed that the miR-101 plasma level was significantly lower in the GC patients than in the healthy volunteers (*P* < 0.0001) (Figure 3A). Furthermore, we used the AUC value and the Youden index [42] to detect any cut-off points that could differentiate cancer patients from healthy volunteers (Figure 3B) and determined the AUC value to be 0.740. The optimal relative expression cut-off point was 8.64, with a sensitivity of 56.3% and a specificity of 82.5%. Our results indicate that miR-101 plasma level can be used to distinguish GC patients from healthy volunteers to a clinically satisfactory degree comparable with that of conventional tumor markers.

### Correlation between miR-101 plasma level and clinicopathological factors in GC patients

We analyzed the correlation between miR-101 plasma level and clinicopathological factors in 128 GC patients undergoing curative gastrectomy (Table 1). The median follow-up period was 39.9 months. A low miR-101 plasma level was significantly correlated with advanced T stages (*P* = 0.0011) and TNM stage (*P* = 0.0072). After gastrectomy, patients with a low miR-101 plasma level more frequently developed peritoneal metastases (*P* = 0.0413) (Table 2).

**Table 1:** Association between plasma miR-101 levels and clinicopathological characteristics in GC patients

		Plasma miR-101 concentration				Univariate
	*n*	High		Low		*P*-value^a^
Total	128	63		65		
Age						0.1423
< 65	36	14	(22%)	22	(34%)	
> 65	92	49	(78%)	43	(66%)	
Sex						0.5596
Male	78	40	(63%)	38	(58%)	
Female	50	23	(37%)	27	(42%)	
pT (TNM)						**0.0011**
T1–2	84	50	(79%)	34	(52%)	
T3–4	44	13	(21%)	31	(48%)	
pN (TNM)						0.2563
N0	79	42	(67%)	37	(57%)	
N1	49	21	(33%)	28	(43%)	
pStage (TNM)						**0.0072**
Stage I/II	99	55	(87%)	44	(68%)	
Stage III/IV	29	8	(23%)	21	(32%)	

^a^Chi-square test. Significant values are in bold.

**Table 2:** Association between plasma miR-101 levels and pattern of recurrence in GC patients with gastrectomy

		Plasma miR-101 concentration				Univariate
	*n*	High		Low		*P*-value^a^
Total	23	9	(14%)	14	(22%)	0.2835
Hematogenous recurrence	5	3	(5%)	2	(3%)	0.6218
Lymphatic recurrence	6	3	(5%)	3	(5%)	0.9687
Peritoneal recurrence	13	3	(5%)	10	(15%)	**0.0413**

^a^Chi-square test. Significant values are in bold.

**Figure 4:**
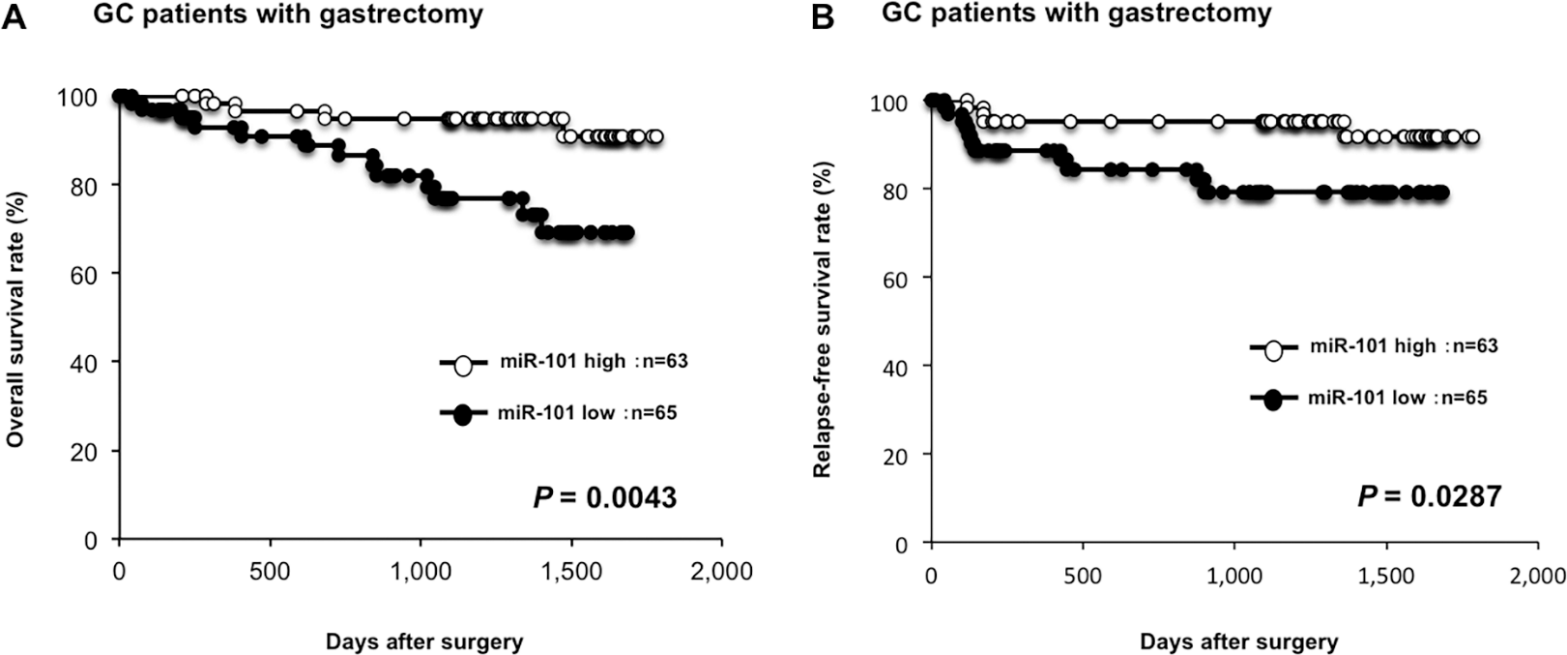
Lower plasma miR-101 level was associated with worse prognosis The prognostic analysis revealed that a low miR-101 plasma level was significantly associated with a worse overall survival rate (*P* = 0.0043) (**A**) and a worse relapse-free survival rate in GC patients with curative gastrectomy (*P* = 0.0287) (**B**).

Moreover, prognostic analysis revealed that a low miR-101 plasma level was significantly associated with a worse overall survival rate (*P* = 0.0043) (Figure 4A) and a worse relapse-free survival rate in GC patients with curative gastrectomy (*P* = 0.0287) (Figure 4B). Univariate and multivariate analyses using the Cox proportional hazards regression model revealed that a low miR-101 level (*P* = 0.0368; hazard ratio, 3.07; 95% confidence interval [CI]: 1.06–11.08) predicted poor prognosis in GC patients independent of TNM stage (Table 3).

**Table 3:** Univariate and multivariate analyses for survival of GC patients following gastrectomy using Cox's proportional hazard model

	Variable	Univariate	Multivariate		
		*P*-value^a^	HR	95% CI	*P*-value^b^
Sex	Male vs. female	0.7389			
Age	≥ 65 vs. 65 <	0.5255			
Tumor location	U vs. LM	0.6220			
Histological type	UD vs. WD	0.6743			
pStage (TNM)	III/IV vs. I/II	**< 0.0001**	14.72	5.13–52.81	**< 0.0001**
Plasma miR-101 expression	High vs. Low	**0.0043**	3.078	1.06–11.08	**0.0368**

^a^Kaplan-Meier method. Significance was determined by the log-rank test. ^b^Multivariate survival analysis was performed using Cox's proportional hazard model. HR: hazard ratio; CI: confidence interval. Significant values are in bold.

### Investigation of the tumor suppressor function of miR-101 in GC cells

**Figure 5:**
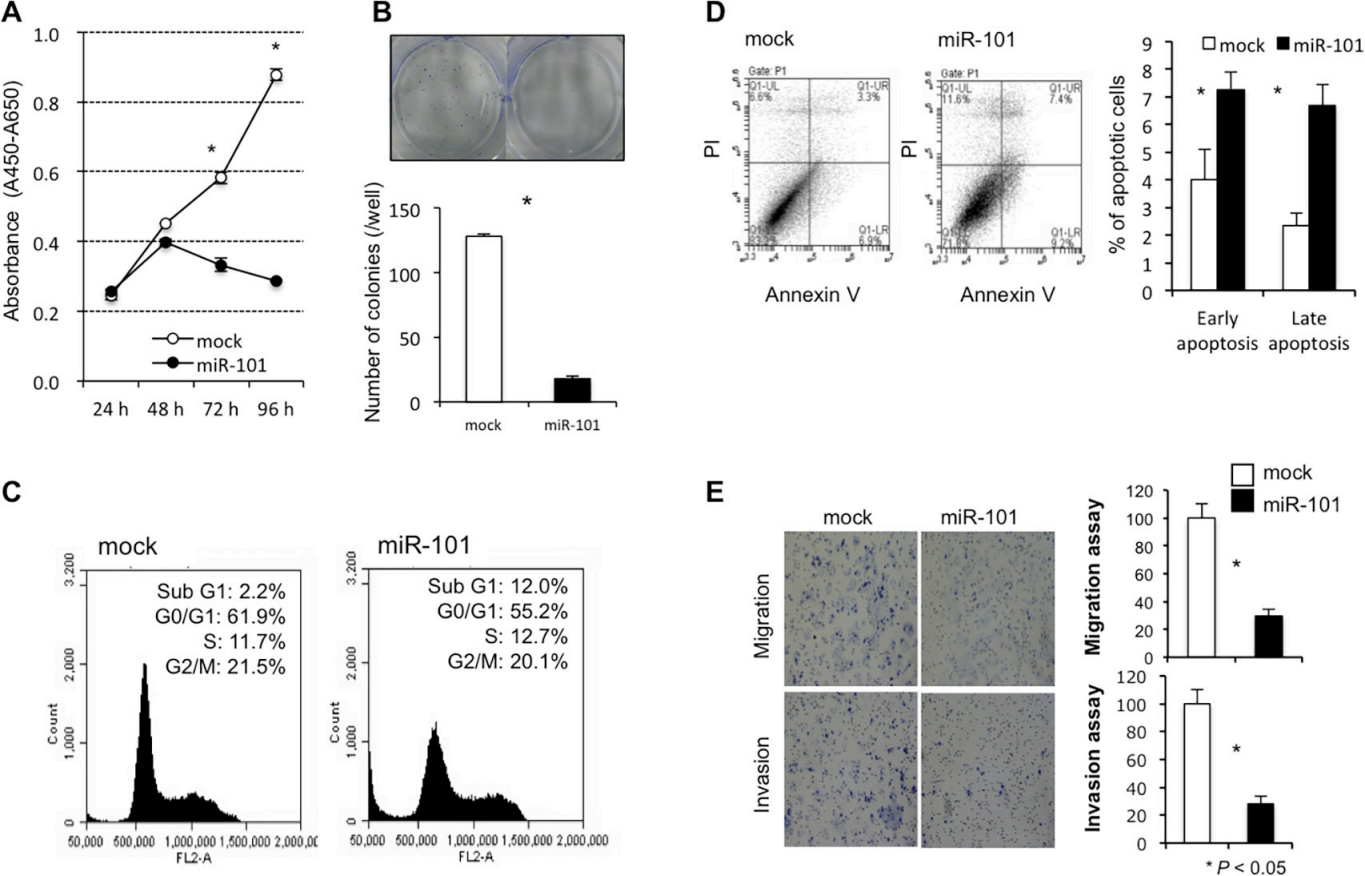
Investigation of the tumor suppressor function of miR-101 in GC cells Cell proliferation was significantly suppressed in GC cells transfected with the miR-101 mimic compared with the controls (**A**). miR-101 overexpression also inhibited colony formation compared with the controls (**B**). The FACS analysis demonstrated that transfecting GC cells with the miR-101 mimic resulted in a greater accumulation of cells in the sub G1 phase compared to transfection with the controls (**C**). The apoptotic cell analysis showed that miR-101 overexpression increased early apoptosis (annexin V-positive/PI-negative) and late apoptosis (annexin V/PI-double positive) at 72 h in GC cells after miR-101 mimic transfection compared to that in GC cells after transfection with the controls (**D**). Transwell migration and invasion assays demonstrated that miR-101 suppressed the ability of GC cells to migrate and invade (**E**).

To investigate the tumor suppressor function of miR-101 in GC cells, we first performed a cell proliferation assay using miRNA mimics to investigate whether miR-101 overexpression would suppress GC cell proliferation. Proliferation was significantly suppressed in MKN45 cells after miR-101 mimic transfection compared with mock transfection (Figure 5A). To confirm the inhibitory effect of miR-101 against anchorage-independent cell growth, colony-formation assays were performed using MKN45 cells. These GC cells were transfected with miR-101 or mock for 2 weeks. The number of colonies was significantly lower in MKN45 cells treated with the miR-101 mimic than in MKN45 cells treated with the mock (Figure 5B). The FACS analysis revealed that transfecting GC cells with the miR-101 mimic induced greater accumulation of sub-G1 phase cells compared with mock transfection (Figure 5C). The apoptotic cell analysis showed that miR-101 overexpression in MKN45 cells increased early apoptosis (annexin V-positive/PI-negative) and late apoptosis (annexin V/PI-double positive) 72 h after miR-101 mimic transfection compared with mock transfection (Figure 5D).

Next, transwell migration and invasion assays were performed to examine the ability of MKN45 cells transfected with miR-101 mimics to move through pores under different conditions. An uncoated membrane was used for migration assays, whereas a Matrigel-coated membrane was used for invasion assays. As seen in Figure 5E, the number of MKN45 cells that migrated into the lower chamber was significantly lower for miR-101 mimic-transfected cells than for mock-transfected cells under both conditions, suggesting that miR-101 suppressed the ability of gastric cancer cells to migrate and invade.

### MCL1 and ZEB1 were direct targets of miR-101 in GC cells

**Figure 6:**
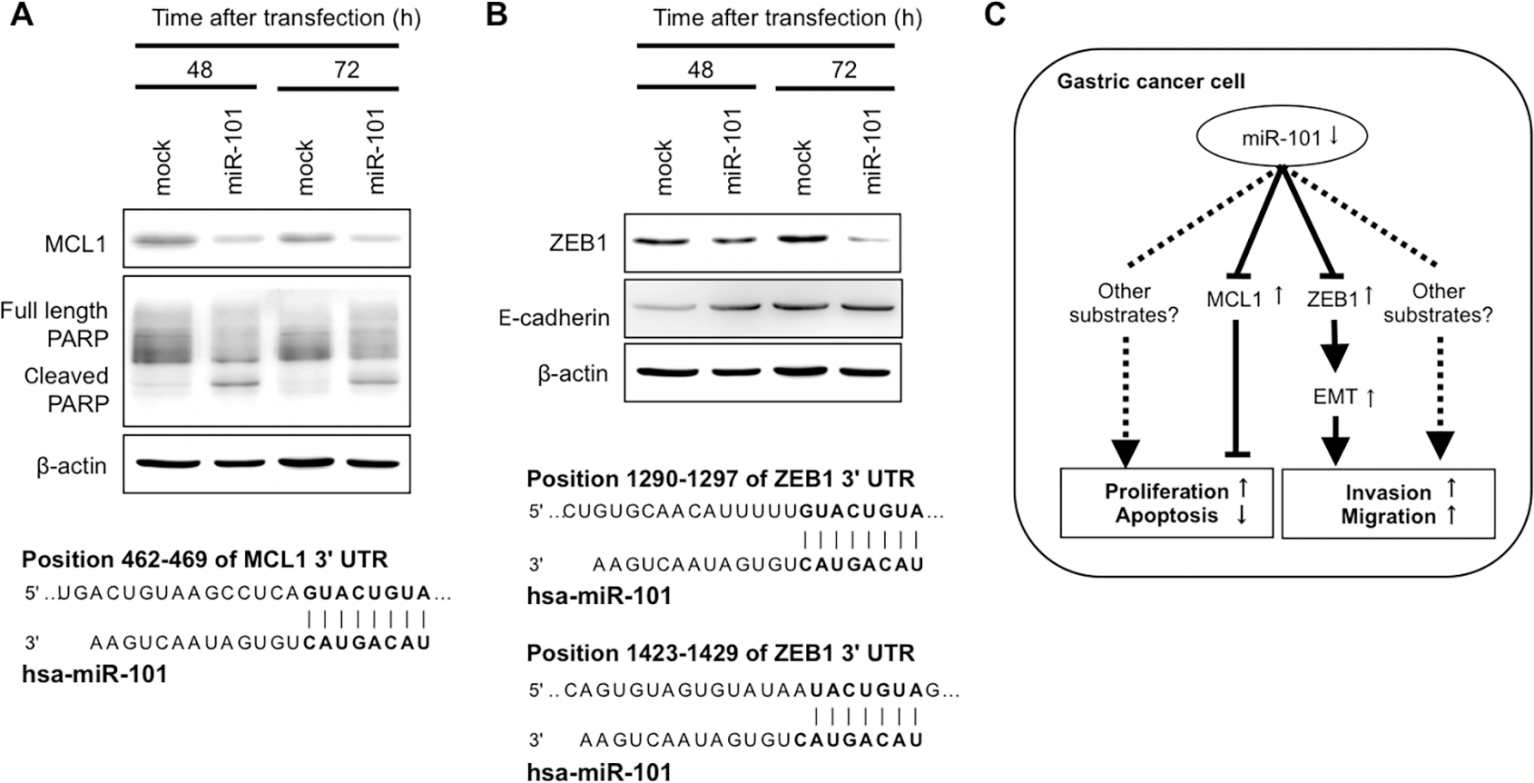
MCL1 and ZEB1 were direct targets of miR-101 in GC cells An *in silico* search (http://www.targetscan.org/) identified MCL1 and ZEB1 as novel target oncogenes of miR-101 in GC. The seed regions of the miR-101 and complementary 3′UTR sequences of MCL1 and ZEB1 are presented in (**A**) and (**B**), respectively. Overexpression of miR-101 inhibited MCL1 protein production (A) and induced the cleavage of PARP, suggesting the induction of apoptosis. Furthermore, miR-101 overexpression inhibited ZEB1 protein production (B). A hypothetical model of miR-101 depletion in gastric cancer cells follows (**C**). Downregulation of miR-101, which inhibits the transcription of the antiapoptotic protein MCL1, could inhibit apoptosis. Furthermore, miR-101 inhibits the transcription of ZEB1, a transcription factor that induces the epithelial-mesenchymal transition, thereby promoting tumor invasion and metastasis.

To investigate whether miR-101 directly regulates target oncogenes, we focused on the *MCL1* and *ZEB1* genes, which were selected as putative targets using TargetScan (http://www.targetscan.org/). MCL1, a member of the Bcl-2 family, is an antiapoptotic protein frequently overexpressed in cancer cells [43]. The seed regions of the miR-101 and complementary *MCL1* 3′UTR sequences are presented in Figure 6A. miR-101 overexpression inhibited the production of MCL1 and induced the cleavage of PARP, suggesting that it induced apoptosis in GC cells.

ZEB1 has been shown to promote the invasiveness of epithelial tumors by repressing E-cadherin promoter and inducing an epithelial-mesenchymal transition (EMT). The seed regions of the miR-101 and complementary *ZEB1* 3′UTR sequences are presented in Figure 6B. miR-101 overexpression inhibited the production of ZEB1 protein and induced the production of E-cadherin protein. These findings suggested that miR-101 directly inhibited the transcription of ZEB1 and suppressed EMT. Figure 6C shows the hypothetical model of the depleted miR-101 in gastric cancer cells.

## DISCUSSION

In the present study, we demonstrated that the tumor suppressor miR-101 was significantly depleted in the plasma of GC patients compared with that of healthy volunteers. The downregulation of plasma miR-101 was related to tumor progression and worse outcomes in GC patients. Moreover, the level of exosomal miR-101 was significantly downregulated in GC patients compared with that in healthy volunteers. These results indicated that miR-101 levels reflect tumor dynamics and that miR-101 may be incorporated into exosomes and released into the plasma as an intercellular transmitter. Additionally, *in vitro* analyses showed that the enforced expression of miR-101 in GC cells induced apoptosis through the regulation of MCL1 and suppressed cell migration and invasion through the regulation of ZEB1. Based on these findings, we consider that miR-101 could be a novel plasma biomarker for cancer detection, monitoring, and prognosis prediction in GC patients. In addition, the restoration of miR-101 plasma level may be a novel treatment strategy for GC patients, and plasma miR-101 level as a biomarker may be used to select treatments, monitor their effects, and predict the possibility of prognostic improvement.

The dynamics and origin of miR-101 circulation in the human body have not yet been elucidated. Recent studies have demonstrated that extracellular miRNAs not only circulate in a stable form, but can also be incorporated into other surrounding and distant recipient cells in which they fulfill distinct functions. Kosaka *et al.* reported that tumor suppressor miRNAs were secreted by normal epithelial cells, and these secretory miRNAs could inhibit growth in cancer cells [24]. Based on these previous studies [17, 19, 29], we hypothesized that healthy cells secrete miR-101 packaged in exosomes into the bloodstream and that these exosomes are delivered via the bloodstream to GC cells. The miR-101 taken up by recipient cells might then serve as an antitumor molecule. During the initial stage of tumorigenesis, the downregulation of tumor suppressor miRNAs in cancer cells may be compensated for by the surrounding healthy cells, which supply exosomes containing these miRNAs. However, once the surrounding cells can no longer meet this demand, the cancer cells progress to an advanced stage. The miR-101 plasma level could therefore be a novel biomarker for GC patients. Additionally, the restoration and maintenance of miR-101 via nucleic acid medicine could be a novel treatment strategy for GC patients.

Concerning the molecular functions of miR-101 in cancers, Varambally *et al.* firstly demonstrated that miR-101 expression was reduced in prostate tumors and miR-101 inhibited the expression of Enhancer of zeste homolog 2 (EZH2), a mammalian histone methyltransferase that contributes to the epigenetic silencing of target genes and regulates the survival and metastasis of cancer cells (Varambally *et al.* [44]. The reduced expression of miR-101 has since been reported in various types of cancer, and several studies have identified the tumor suppressor functions of miR-101. One crucial function of miR-101 is the inhibition of oncogenes such as EZH2 [45–50], ROCK2 [51], COX-2 [52–55], MCL1 [56, 57], mTOR [58], SOCS2 [59], and VEGF-C [60, 61]. Especially in GC, Liu et al. reported that miR-101 could inhibit angiogenesis by down-regulating VEGF-C expression [60], and Riquelme et al. reported that miR-101 suppressed cell proliferation, migration, and invasion in gastric cancer cells through regulation of the PI3K/AKT/mTOR pathway [58]. In this study, our results showed that miR-101 could induce apoptosis by targeting the antiapoptosis protein MCL1, which is highly upregulated and correlated with poor prognosis in GC patients [43]. The results are consistent with the previous report in breast cancer [56]. Furthermore, our results newly demonstrated that miR-101 could inhibit EMT, as well as cell invasion and migration, through the regulation of ZEB1 in GC cells. In addition to these functions of miR-101 in cancer cells, interestingly, previous studies reported that miR-101 was significantly down-regulated in *H. pylori*-positive gastric mucosa, and miR-101 expression was decreased along with the severity of gastritis, such as superficial gastritis, atrophic gastritis, and metaplasia. Furthermore, miR-101 suppressed cell growth in *H. pylori*-related GC [59]. These findings suggest that the restoration of miR-101 may have the potential to prevent tumorigenesis in pre-cancerous lesions of atrophic gastritis due to *H. pylori*.

Although miR-101 has demonstrated promising tumor suppressor functions in several cancers, including GC, few studies have investigated the potential utility of circulating miR-101 as a liquid biopsy biomarker and therapeutic target.

In recent years, several researchers have conducted studies on therapeutic miRNA-based drugs using synthetic miRNA mimics. The administration of tumor suppressor miRNA mimics still carries the potential risk of inducing unexpected adverse physiological effects because miRNAs can regulate multiple genes affecting various biological functions. In this study, we focused on the depletion of tumor suppressor miRNAs in GC patients and demonstrated that the restoration of miR-101 to GC cells could be a novel anticancer treatment for GC. We believe that the restoration of tumor suppressor miRNAs, which are abundantly detected in the plasma of healthy individuals, may be a novel strategy for minimizing various physiological risks in systemic administration. Furthermore, the systemic administration and delivery of miR-101 for GC patients may be an ideal treatment because its effect and the possibility of prognosis improvement can be monitored in a repeatable manner by measuring plasma miR-101 level.

This report is the first to demonstrate that the tumor suppressor miR-101, which is depleted in the plasma of GC patients, can serve as both a plasma biomarker and a therapeutic target for GC. However, the present study had the limitation of being a relatively small retrospective cohort study from a single institute. Therefore, further study with a large cohort or prospective clinical trial with longer follow-up periods is needed to validate these results. Furthermore, many issues must still be addressed before these findings can be translated into a clinically useful biomarker and treatment agent for GC patients. Detailed examinations of the physiological effects of miR-101 are required to assess its safeness for systemic administration, and validation of the tumor suppressor functions of miR-101 *in vivo* is necessary for its clinical use. Moreover, further development of miRNA delivery systems is needed to overcome hurdles such as cellular uptake and bloodstream stability. These studies are currently under evaluation. Furthermore, tumor suppressor miRNAs with more powerful anticancer effects could be identified by examining tumor suppressor miRNAs depleted in the plasma of patients with various cancer types using strategies such as microarray analysis, next-generation sequencing, or digital PCR-based approaches. These strategies are currently under evaluation and will likely be reported upon in the near future.

## MATERIALS AND METHODS

### Patients and samples

All experimental methods were conducted in accordance with the relevant guidelines and regulations. Written informed consent was obtained from all patients to use their samples for research purposes, and the study was approved by the institutional review boards of the Kyoto Prefectural University of Medicine. A total of 128 plasma samples from GC patients who underwent gastrectomy at our institution between June 2010 and December 2014 were collected, along with 80 samples from healthy volunteers. Patients’ clinical characteristics and compliance with REMARK guidelines are reported in Supplementary Tables 1 and 2, respectively. All of the patients enrolled in this study were underwent curative gastrectomy, and all of the tumors were pathologically diagnosed as gastric adenocarcinoma. The healthy volunteers included medical personnel and patients with benign diseases such as cholecystolithiasis and inguinal herniation. These patients underwent medical examinations, including computed tomography and endoscopy, and were shown not to have any pancreatic or cancerous diseases. Tumor stages were assessed according to the Union for International Cancer Control classification system [62].

Peripheral blood (7 ml) was obtained from each patient at the time of diagnosis or before surgery, as well as from the healthy volunteers. The blood was transferred into sodium heparin tubes (BD Vacutainer, Franklin Lakes, NJ) and immediately subjected to the three-spin protocol (1500 rpm for 30 min, 3000 rpm for 5 min, and 4500 rpm for 5 min) to prevent contamination of the cellular nucleic acids. Plasma was stored at −80°C until further processing. Histological evaluations were performed for tissues adjacent to the specimens according to the criteria of the World Health Organization. In all cases, two pathologists agreed with the pathological observations and confirmed the diagnoses.

### RNA extraction

Total RNA was extracted from 400 μl of plasma using a mirVana PARIS Kit (Ambion, Austin, TX) and finally eluted into 100 μl of preheated (95°C) Elution Solution according to the manufacturer’s protocol. The reason why a volume of 400 μl of plasma was used as the common denominator in each microarray analysis is that there was no definite internal control in the plasma miRNA analyses, as shown in our previous studies [27–32, 63]. Total RNA was also extracted from four 15-μm-thick slices of formalin-fixed and paraffin-embedded tissue (for a total of 60 μm in thickness) using a RecoverAll Total Nucleic Acid Isolation Kit (Ambion) and then eluted into 60 μl of Elution Solution according to the manufacturer’s protocol.

### Quantification of miRNA by qRT-PCR

The miRNAs were quantified by qRT-PCR using a Human TaqMan MicroRNA Assay Kit (Applied Biosystems, Foster City, CA). The reverse transcription reaction was performed using a TaqMan MicroRNA Reverse Transcription Kit (Applied Biosystems). qPCR was run on a StepOnePlus PCR system (Applied Biosystems), and cycle threshold (Ct) values were calculated with StepOne Software v2.0 (Applied Biosystems).

As previously reported [13], we used an approach for data normalization based on spiking the samples with a synthetic RNA oligonucleotide, cel-miR-39, which does not exist in the human genome. *C. elegans* cel-miR-39 was purchased as a custom-made RNA oligonucleotide (*Qiagen*, Valencia, CA). The oligo used for spiking, as a mixture of 25 fmol of oligonucleotide and water in a total volume of 5 µl, was introduced after the addition of 2X Denaturing Solution (Ambion) to the plasma sample to avoid degradation by endogenous plasma RNases. As a control for each RNA sample, cel-miR-39 was used for the TaqMan qRT-PCR assays (Applied Biosystems) as described above. We normalized the data across samples using the 2^−ΔΔCt^ method relative to cel-miR-39. However, the expression of miRNAs from tissue samples and cultured cells was normalized using the 2^−ΔΔCt^ method relative to U6 small nuclear RNA (RNU6B).

### Culture of GC cell lines

The GC cell lines Kato-III, NUGC4, MKN45, and MKN74 were purchased from RIKEN Cell Bank (Tsukuba, Japan) and cultured in either Roswell Park Memorial Institute 1640 medium (Sigma, St. Louis, MO) or Dulbecco’s Modified Eagle Medium (Nacalai, Japan) supplemented with 10% fetal bovine serum (Trace Scientific, Melbourne, Australia). All cells were cultured in 5% carbon dioxide at 37°C in a humidified chamber.

### Transfection of GC cells with miRNA mimics

For the overexpression of miR-101, an miR-101 mimic (Assay ID: MC11414) selected from the mirVana miRNA mimic panel (Ambion) was used to transfect the MKN45 cells at a final concentration of 12 μM using Lipofectamine RNAiMAX (Invitrogen) according to the manufacturer’s instructions. After 72 h, the overexpression of miR-101 was confirmed by qRT-PCR using a Human TaqMan MicroRNA Assay Kit (Applied Biosystems).

### Proliferation assay and cell cycle analysis

To evaluate cell growth function, the number of viable cells was assessed at various time points after transfection using the colorimetric water-soluble tetrazolium salt assay (Cell Counting Kit 8; Dojindo Laboratories, Kumamoto, Japan). Cell viability was determined by measuring the optical density at 450 nm. The cell cycle was evaluated 72 h after transfection using fluorescence-activated cell sorting (FACS), as described elsewhere [64]. For the FACS analysis, harvested cells were fixed in 70% cold ethanol and treated with RNase A and propidium iodide. Samples were analyzed on a Becton Dickinson Accuri™ C6 Flow Cytometer (Becton Dickinson, San Jose, CA, USA).

### Colony-formation assays

The miR-101 or mock was transfected into GC cells. The expression of the miR-101 mimic in transfected cells was confirmed by qRT-PCR. After 2 weeks of incubation, the cells were fixed with 100% methanol and stained with crystal violet.

### Apoptotic cell analysis

At 72 h after transfection, the miRNA mimic–transfected cells were harvested and stained with fluorescein isothiocyanate–conjugated annexin V and phosphatidylinositol using an Annexin V Kit (Beckman Coulter, Brea, CA). A Becton Dickinson Accuri™ C6 Flow Cytometer was used to analyze the proportion of apoptotic cells.

### Transwell migration and invasion assays

Transwell migration and invasion assays were conducted in 24-well modified Boyden chambers (Transwell chambers, BD Transduction, Franklin Lakes, NJ). The upper surface of 6.4-mm-diameter filters with 8-µm pores was precoated with (invasion assay) or without (migration assay) Matrigel (BD Transduction). The miRNA mimic transfectants (5 × 10^5^ cells per well) were transferred into the upper chamber. Following 22 h of incubation, the migrated or invasive cells on the lower surface of the filters were fixed and stained with Diff-Quik stain (Sysmex, Kobe, Japan), and stained cell nuclei were counted directly in triplicate.

### Western blot analysis

Anti-ACTB, anti-ZEB1, anti-E-cadherin, anti-MCL1, and anti-PARP antibodies were purchased from Cell Signaling Technology (Cell Signaling Technology, USA). The cells were lysed, and their proteins were extracted using M-PER^®^ Mammalian Protein Extraction Reagent (Thermo Scientific, USA)

### Isolation of exosomes from plasma

Exosomes were extracted from the plasma using a miRCURY Exosomes Isolation Kit – Serum and Plasma (Exiqon). Thrombin was added to the plasma, and the supernatants were collected after centrifugation at 10,000 g for 5 min. Precipitation buffer was added to the supernatants, and exosome pellets were collected by centrifugation at 500 g for 5 min after incubation for 60 min at 4°C.

### Statistical analysis

For the miRNA array–based analyses, the signal intensity ratio of each plasma miRNA was calculated as the signal intensity ratio of GC patients to healthy volunteers. The Mann–Whitney *U* test and the *t*-test for unpaired data were performed to compare plasma and tissue sample data. The Wilcoxon test was used to compare the paired plasma samples obtained before and 1 month after pancreatectomy, as well as the paired tumor and normal tissue samples. The chi-square test or Fisher’s exact probability test were used to evaluate correlations between the plasma miRNA levels and clinicopathological factors. A *P*-value < 0.05 was considered statistically significant.

Receiver-operating characteristic (ROC) curves and area under the ROC curve (AUC) values were used to assess the feasibility of using plasma miRNA levels as a diagnostic tool for detecting GC. The Youden index was used to determine the cut-off value for the plasma miRNA levels [42]. For the survival rate analysis, Kaplan-Meier survival curves were constructed for groups based on univariate predictors, and differences between the groups were analyzed with the log-rank test or the Wilcoxon test. Univariate and multivariate survival analyses were performed using the likelihood ratio test of the stratified Cox proportional hazards model. A *P*-value < 0.05 was considered statistically significant.

## SUPPLEMENTARY MATERIALS TABLES



## Abbreviations

GC: gastric cancer
miRNA: microRNA
EMT: epithelial-mesenchymal transition
ZEB1: Zinc finger E-box-binding homeobox 1
HR: hazard ratio
CI: confidence interval

## References

[1] Torre LA, Bray F, Siegel RL, Ferlay J, Lortet-Tieulent J, Jemal A (2015). Global cancer statistics, 2012. CA Cancer J Clin.

[2] Van Cutsem E, Sagaert X, Topal B, Haustermans K, Prenen H (2016). Gastric cancer. Lancet.

[3] Bang YJ, Van Cutsem E, Feyereislova A, Chung HC, Shen L, Sawaki A, Lordick F, Ohtsu A, Omuro Y, Satoh T, Aprile G, Kulikov E, Hill J, ToGA Trial Investigators (2010). Trastuzumab in combination with chemotherapy versus chemotherapy alone for treatment of HER2-positive advanced gastric or gastro-oesophageal junction cancer (ToGA): a phase 3, open-label, randomised controlled trial. Lancet.

[4] Lordick F, Kang YK, Chung HC, Salman P, Oh SC, Bodoky G, Kurteva G, Volovat C, Moiseyenko VM, Gorbunova V, Park JO, Sawaki A, Celik I, Arbeitsgemeinschaft Internistische Onkologie and EXPAND Investigators (2013). Capecitabine and cisplatin with or without cetuximab for patients with previously untreated advanced gastric cancer (EXPAND): a randomised, open-label phase 3 trial. Lancet Oncol.

[5] Waddell T, Chau I, Cunningham D, Gonzalez D, Okines AF, Okines C, Wotherspoon A, Saffery C, Middleton G, Wadsley J, Ferry D, Mansoor W, Crosby T (2013). Epirubicin, oxaliplatin, and capecitabine with or without panitumumab for patients with previously untreated advanced oesophagogastric cancer (REAL3): a randomised, open-label phase 3 trial. Lancet Oncol.

[6] Satoh T, Xu RH, Chung HC, Sun GP, Doi T, Xu JM, Tsuji A, Omuro Y, Li J, Wang JW, Miwa H, Qin SK, Chung IJ (2014). Lapatinib plus paclitaxel versus paclitaxel alone in the second-line treatment of HER2-amplified advanced gastric cancer in Asian populations: TyTAN—a randomized, phase III study. J Clin Oncol.

[7] Fuchs CS, Tomasek J, Yong CJ, Dumitru F, Passalacqua R, Goswami C, Safran H, Dos Santos LV, Aprile G, Ferry DR, Melichar B, Tehfe M, Topuzov E, REGARD Trial Investigators (2014). Ramucirumab monotherapy for previously treated advanced gastric or gastro-oesophageal junction adenocarcinoma (REGARD): an international, randomised, multicentre, placebo-controlled, phase 3 trial. Lancet.

[8] Lee RC, Feinbaum RL, Ambros V (1993). The C. elegans heterochronic gene lin-4 encodes small RNAs with antisense complementarity to lin-14. Cell.

[9] He L, Thomson JM, Hemann MT, Hernando-Monge E, Mu D, Goodson S, Powers S, Cordon-Cardo C, Lowe SW, Hannon GJ, Hammond SM (2005). A microRNA polycistron as a potential human oncogene. Nature.

[10] Calin GA, Croce CM (2006). MicroRNA signatures in human cancers. Nat Rev Cancer.

[11] He L, He X, Lim LP, de Stanchina E, Xuan Z, Liang Y, Xue W, Zender L, Magnus J, Ridzon D, Jackson AL, Linsley PS, Chen C (2007). A microRNA component of the p53 tumour suppressor network. Nature.

[12] Lu J, Getz G, Miska EA, Alvarez-Saavedra E, Lamb J, Peck D, Sweet-Cordero A, Ebert BL, Mak RH, Ferrando AA, Downing JR, Jacks T, Horvitz HR, Golub TR (2005). MicroRNA expression profiles classify human cancers. Nature.

[13] Mitchell PS, Parkin RK, Kroh EM, Fritz BR, Wyman SK, Pogosova-Agadjanyan EL, Peterson A, Noteboom J, O’Briant KC, Allen A, Lin DW, Urban N, Drescher CW (2008). Circulating microRNAs as stable blood-based markers for cancer detection. Proc Natl Acad Sci USA.

[14] Chen X, Ba Y, Ma L, Cai X, Yin Y, Wang K, Guo J, Zhang Y, Chen J, Guo X, Li Q, Li X, Wang W (2008). Characterization of microRNAs in serum: a novel class of biomarkers for diagnosis of cancer and other diseases. Cell Res.

[15] Filipowicz W, Bhattacharyya SN, Sonenberg N (2008). Mechanisms of post-transcriptional regulation by microRNAs: are the answers in sight?. Nat Rev Genet.

[16] Ichikawa D, Komatsu S, Konishi H, Otsuji E (2012). Circulating microRNA in digestive tract cancers. Gastroenterology.

[17] Arroyo JD, Chevillet JR, Kroh EM, Ruf IK, Pritchard CC, Gibson DF, Mitchell PS, Bennett CF, Pogosova-Agadjanyan EL, Stirewalt DL, Tait JF, Tewari M (2011). Argonaute2 complexes carry a population of circulating microRNAs independent of vesicles in human plasma. Proc Natl Acad Sci USA.

[18] Vickers KC, Palmisano BT, Shoucri BM, Shamburek RD, Remaley AT (2011). MicroRNAs are transported in plasma and delivered to recipient cells by high-density lipoproteins. Nat Cell Biol.

[19] Kosaka N, Iguchi H, Ochiya T (2010). Circulating microRNA in body fluid: a new potential biomarker for cancer diagnosis and prognosis. Cancer Sci.

[20] Hasselmann DO, Rappl G, Tilgen W, Reinhold U (2001). Extracellular tyrosinase mRNA within apoptotic bodies is protected from degradation in human serum. Clin Chem.

[21] Cocucci E, Racchetti G, Meldolesi J (2009). Shedding microvesicles: artefacts no more. Trends Cell Biol.

[22] Schwarzenbach H, Hoon DS, Pantel K (2011). Cell-free nucleic acids as biomarkers in cancer patients. Nat Rev Cancer.

[23] Valadi H, Ekström K, Bossios A, Sjöstrand M, Lee JJ, Lötvall JO (2007). Exosome-mediated transfer of mRNAs and microRNAs is a novel mechanism of genetic exchange between cells. Nat Cell Biol.

[24] Kosaka N, Iguchi H, Yoshioka Y, Takeshita F, Matsuki Y, Ochiya T (2010). Secretory mechanisms and intercellular transfer of microRNAs in living cells. J Biol Chem.

[25] Skog J, Würdinger T, van Rijn S, Meijer DH, Gainche L, Sena-Esteves M, Curry WT, Carter BS, Krichevsky AM, Breakefield XO (2008). Glioblastoma microvesicles transport RNA and proteins that promote tumour growth and provide diagnostic biomarkers. Nat Cell Biol.

[26] Rechavi O, Erlich Y, Amram H, Flomenblit L, Karginov FV, Goldstein I, Hannon GJ, Kloog Y (2009). Cell contact-dependent acquisition of cellular and viral nonautonomously encoded small RNAs. Genes Dev.

[27] Tsujiura M, Ichikawa D, Komatsu S, Shiozaki A, Takeshita H, Kosuga T, Konishi H, Morimura R, Deguchi K, Fujiwara H, Okamoto K, Otsuji E (2010). Circulating microRNAs in plasma of patients with gastric cancers. Br J Cancer.

[28] Morimura R, Komatsu S, Ichikawa D, Takeshita H, Tsujiura M, Nagata H, Konishi H, Shiozaki A, Ikoma H, Okamoto K, Ochiai T, Taniguchi H, Otsuji E (2011). Novel diagnostic value of circulating miR-18a in plasma of patients with pancreatic cancer. Br J Cancer.

[29] Komatsu S, Ichikawa D, Takeshita H, Konishi H, Nagata H, Hirajima S, Kawaguchi T, Arita T, Shiozaki A, Fujiwara H, Okamoto K, Otsuji E (2012). Prognostic impact of circulating miR-21 and miR-375 in plasma of patients with esophageal squamous cell carcinoma. Expert Opin Biol Ther.

[30] Kawaguchi T, Komatsu S, Ichikawa D, Morimura R, Tsujiura M, Konishi H, Takeshita H, Nagata H, Arita T, Hirajima S, Shiozaki A, Ikoma H, Okamoto K (2013). Clinical impact of circulating miR-221 in plasma of patients with pancreatic cancer. Br J Cancer.

[31] Hirajima S, Komatsu S, Ichikawa D, Takeshita H, Konishi H, Shiozaki A, Morimura R, Tsujiura M, Nagata H, Kawaguchi T, Arita T, Kubota T, Fujiwara H (2013). Clinical impact of circulating miR-18a in plasma of patients with oesophageal squamous cell carcinoma. Br J Cancer.

[32] Komatsu S, Ichikawa D, Hirajima S, Kawaguchi T, Miyamae M, Okajima W, Ohashi T, Arita T, Konishi H, Shiozaki A, Fujiwara H, Okamoto K, Yagi N, Otsuji E (2014). Plasma microRNA profiles: identification of miR-25 as a novel diagnostic and monitoring biomarker in oesophageal squamous cell carcinoma. Br J Cancer.

[33] Komatsu S, Ichikawa D, Miyamae M, Kawaguchi T, Morimura R, Hirajima S, Okajima W, Ohashi T, Imamura T, Konishi H, Shiozaki A, Ikoma H, Okamoto K (2015). Malignant potential in pancreatic neoplasm; new insights provided by circulating miR-223 in plasma. Expert Opin Biol Ther.

[34] Miyamae M, Komatsu S, Ichikawa D, Kawaguchi T, Hirajima S, Okajima W, Ohashi T, Imamura T, Konishi H, Shiozaki A, Morimura R, Ikoma H, Ochiai T (2015). Plasma microRNA profiles: identification of miR-744 as a novel diagnostic and prognostic biomarker in pancreatic cancer. Br J Cancer.

[35] Tsujiura M, Komatsu S, Ichikawa D, Shiozaki A, Konishi H, Takeshita H, Moriumura R, Nagata H, Kawaguchi T, Hirajima S, Arita T, Fujiwara H, Okamoto K, Otsuji E (2015). Circulating miR-18a in plasma contributes to cancer detection and monitoring in patients with gastric cancer. Gastric Cancer.

[36] Kawaguchi T, Komatsu S, Ichikawa D, Tsujiura M, Takeshita H, Hirajima S, Miyamae M, Okajima W, Ohashi T, Imamura T, Kiuchi J, Konishi H, Shiozaki A (2016). Circulating MicroRNAs: A Next-Generation Clinical Biomarker for Digestive System Cancers. Int J Mol Sci.

[37] Komatsu S, Ichikawa D, Kawaguchi T, Miyamae M, Okajima W, Ohashi T, Imamura T, Kiuchi J, Konishi H, Shiozaki A, Fujiwara H, Okamoto K, Otsuji E (2016). Circulating miR-21 as an independent predictive biomarker for chemoresistance in esophageal squamous cell carcinoma. Am J Cancer Res.

[38] Komatsu S, Ichikawa D, Kawaguchi T, Takeshita H, Miyamae M, Ohashi T, Okajima W, Imamura T, Kiuchi J, Arita T, Konishi H, Shiozaki A, Fujiwara H (2016). Plasma microRNA profiles: identification of miR-23a as a novel biomarker for chemoresistance in esophageal squamous cell carcinoma. Oncotarget.

[39] Okajima W, Komatsu S, Ichikawa D, Miyamae M, Kawaguchi T, Hirajima S, Ohashi T, Imamura T, Kiuchi J, Arita T, Konishi H, Shiozaki A, Moriumura R (2016). Circulating microRNA profiles in plasma: identification of miR-224 as a novel diagnostic biomarker in hepatocellular carcinoma independent of hepatic function. Oncotarget.

[40] Komatsu S, Ichikawa D, Takeshita H, Tsujiura M, Morimura R, Nagata H, Kosuga T, Iitaka D, Konishi H, Shiozaki A, Fujiwara H, Okamoto K, Otsuji E (2011). Circulating microRNAs in plasma of patients with oesophageal squamous cell carcinoma. Br J Cancer.

[41] Kosaka N, Iguchi H, Yoshioka Y, Hagiwara K, Takeshita F, Ochiya T (2012). Competitive interactions of cancer cells and normal cells via secretory microRNAs. J Biol Chem.

[42] Akobeng AK (2007). Understanding diagnostic tests 3: Receiver operating characteristic curves. Acta paediatrica.

[43] Likui W, Qun L, Wanqing Z, Haifeng S, Fangqiu L, Xiaojun L (2009). Prognostic role of myeloid cell leukemia-1 protein (Mcl-1) expression in human gastric cancer. J Surg Oncol.

[44] Varambally S, Cao Q, Mani RS, Shankar S, Wang X, Ateeq B, Laxman B, Cao X, Jing X, Ramnarayanan K, Brenner JC, Yu J, Kim JH (2008). Genomic loss of microRNA-101 leads to overexpression of histone methyltransferase EZH2 in cancer. Science.

[45] Friedman JM, Liang G, Liu CC, Wolff EM, Tsai YC, Ye W, Zhou X, Jones PA (2009). The putative tumor suppressor microRNA-101 modulates the cancer epigenome by repressing the polycomb group protein EZH2. Cancer Res.

[46] Sachdeva M, Wu H, Ru P, Hwang L, Trieu V, Mo YY (2011). MicroRNA-101-mediated Akt activation and estrogen-independent growth. Oncogene.

[47] Banerjee R, Mani RS, Russo N, Scanlon CS, Tsodikov A, Jing X, Cao Q, Palanisamy N, Metwally T, Inglehart RC, Tomlins S, Bradford C, Carey T (2011). The tumor suppressor gene rap1GAP is silenced by miR-101-mediated EZH2 overexpression in invasive squamous cell carcinoma. Oncogene.

[48] Wang HJ, Ruan HJ, He XJ, Ma YY, Jiang XT, Xia YJ, Ye ZY, Tao HQ (2010). MicroRNA-101 is down-regulated in gastric cancer and involved in cell migration and invasion. Eur J Cancer.

[49] Carvalho J, van Grieken NC, Pereira PM, Sousa S, Tijssen M, Buffart TE, Diosdado B, Grabsch H, Santos MA, Meijer G, Seruca R, Carvalho B, Oliveira C (2012). Lack of microRNA-101 causes E-cadherin functional deregulation through EZH2 up-regulation in intestinal gastric cancer. J Pathol.

[50] Chen DL, Ju HQ, Lu YX, Chen LZ, Zeng ZL, Zhang DS, Luo HY, Wang F, Qiu MZ, Wang DS, Xu DZ, Zhou ZW, Pelicano H (2016). Long non-coding RNA XIST regulates gastric cancer progression by acting as a molecular sponge of miR-101 to modulate EZH2 expression. J Exp Clin Cancer Res.

[51] Ye Z, Yin S, Su Z, Bai M, Zhang H, Hei Z, Cai S (2016). Downregulation of miR-101 contributes to epithelial-mesenchymal transition in cisplatin resistance of NSCLC cells by targeting ROCK2. Oncotarget.

[52] Lin C, Huang F, Shen G, Yiming A (2015). MicroRNA-101 regulates the viability and invasion of cervical cancer cells. Int J Clin Exp Pathol.

[53] He XP, Shao Y, Li XL, Xu W, Chen GS, Sun HH, Xu HC, Xu X, Tang D, Zheng XF, Xue YP, Huang GC, Sun WH (2012). Downregulation of miR-101 in gastric cancer correlates with cyclooxygenase-2 overexpression and tumor growth. FEBS J.

[54] Strillacci A, Griffoni C, Sansone P, Paterini P, Piazzi G, Lazzarini G, Spisni E, Pantaleo MA, Biasco G, Tomasi V (2009). MiR-101 downregulation is involved in cyclooxygenase-2 overexpression in human colon cancer cells. Exp Cell Res.

[55] Gong J, Chu Y, Xu M, Huo J, Lv L (2016). Esophageal squamous cell carcinoma cell proliferation induced by exposure to low concentration of cigarette smoke extract is mediated via targeting miR-101-3p/COX-2 pathway. Oncol Rep.

[56] Liu X, Tang H, Chen J, Song C, Yang L, Liu P, Wang N, Xie X, Lin X, Xie X (2015). MicroRNA-101 inhibits cell progression and increases paclitaxel sensitivity by suppressing MCL-1 expression in human triple-negative breast cancer. Oncotarget.

[57] Su H, Yang JR, Xu T, Huang J, Xu L, Yuan Y, Zhuang SM (2009). MicroRNA-101, down-regulated in hepatocellular carcinoma, promotes apoptosis and suppresses tumorigenicity. Cancer Res.

[58] Riquelme I, Tapia O, Leal P, Sandoval A, Varga MG, Letelier P, Buchegger K, Bizama C, Espinoza JA, Peek RM, Araya JC, Roa JC (2016). miR-101-2, miR-125b-2 and miR-451a act as potential tumor suppressors in gastric cancer through regulation of the PI3K/AKT/mTOR pathway. Cell Oncol (Dordr).

[59] Zhou X, Xia Y, Li L, Zhang G (2015). MiR-101 inhibits cell growth and tumorigenesis of Helicobacter pylori related gastric cancer by repression of SOCS2. Cancer Biol Ther.

[60] Liu HT, Xing AY, Chen X, Ma RR, Wang YW, Shi DB, Zhang H, Li P, Chen HF, Li YH, Gao P (2015). MicroRNA-27b, microRNA-101 and microRNA-128 inhibit angiogenesis by down-regulating vascular endothelial growth factor C expression in gastric cancers. Oncotarget.

[61] Liu Z, Wang J, Mao Y, Zou B, Fan X (2016). MicroRNA-101 suppresses migration and invasion via targeting vascular endothelial growth factor-C in hepatocellular carcinoma cells. Oncol Lett.

[62] Sobin LH, Compton CC (2010). TNM seventh edition: what’s new, what’s changed: communication from the International Union Against Cancer and the American Joint Committee on Cancer. Cancer.

[63] Konishi H, Ichikawa D, Komatsu S, Shiozaki A, Tsujiura M, Takeshita H, Morimura R, Nagata H, Arita T, Kawaguchi T, Hirashima S, Fujiwara H, Okamoto K, Otsuji E (2012). Detection of gastric cancer-associated microRNAs on microRNA microarray comparing pre- and post-operative plasma. Br J Cancer.

[64] Komatsu S, Imoto I, Tsuda H, Kozaki KI, Muramatsu T, Shimada Y, Aiko S, Yoshizumi Y, Ichikawa D, Otsuji E, Inazawa J (2009). Overexpression of SMYD2 relates to tumor cell proliferation and malignant outcome of esophageal squamous cell carcinoma. Carcinogenesis.

