# Supplementary material for: Low plasma levels of miR-101 are associated with tumor progression in gastric cancer
Source: Oncotarget. 2017 Sep 13;8(63):106538–50. doi: 10.18632/oncotarget.20860 (PMC5739754; doi:10.18632/oncotarget.20860)
Supplement: Supplementary file 1 [file oncotarget-08-106538-s001.pdf]

Low plasma levels of miR-101 are associated with tumor progression in gastric cancer

SUPPLEMENTARY MATERIALS

Supplementary Table 1: Patient characteristics

	**All patients (*n* = 128)**
**Age, median (range)**	69 (33–89)
**Sex, *n* (%)**	
Male	78 (60.94)
Female	50 (39.06)
**Stage at diagnosis, *n* (%)**	
I	75 (58.59)
II	24 (18.75)
III	29 (22.66)
**Site of primary tumor, *n* (%)**	
Upper	37 (29.13)
Middle	55 (43.31)
Lower	30 (23.62)
Whole	5 (3.94)
***T* stage, *n* (%)**	
T1	66 (51.56)
T2	18 (14.06)
T3	23 (17.97)
T4	21 (16.41)
***N* stage, *n* (%)**	
N0	79 (62.70)
N1	19 (15.08)
N2	11 (8.73)
N3	17 (13.49)
**Plasma miR-101 level, *n* (%)**	
Low	65 (50.78)
High	63 (49.22)

Supplementary Table 2: Compliance with REMARK guidelines

**Introduction**	
	Marker analyzed is the miR-101 plasma level.
	Objectives; to determine whether plasma miR-101 is predictive for prognosis in gastric cancer patients.
	Hypotheses; Low plasma level of miR-101 is associated with poor outcomes.
**Materials and Methods**	
- Patients	Primary gastric cancer patients treated with curative gastrectomy.
- Specimen characteristics	A total of 128 plasma samples from gastric cancer patients who underwent gastrectomy at our institution, and 80 samples of healthy volunteers included medical personnel and patients with benign diseases such as cholecystolithiasis and inguinal herniation.
- Assay method	The plasma miRNAs were quantified by qRT-PCR. Detailed protocol described in Materials and Methods section.
- Study design	Retrospective analysis of plasma samples from patients collected between June 2010 and December 2014. The median follow-up was 35.9 months.
	Clinical end-point, overall survival (OS).
	Sample size determined by the availability of samples.
- Statistical methods	Survival curves of patients stratified according to the miR-101 expression were drawn by Kaplan-Meier estimates and compared by log rank test.
	Multivariate analysis of OS and RFS, with stepwise variable selection, was conducted by Cox's proportional hazard regression model to assess the independent predictive value of plasma level of miR-101 .
	Relationship between plasma miR-101 level and clinic-pathological characteristics was examined using the $\chi 2$ method.
**Results**	
- Data	Patient's characteristics reported in Supplementary Table 1.
- Analysis and presentation	Correlation between plasma miR-101 expression and clinic-pathologic characteristic displayed in Table 1.
	Kaplan–Meier survival curves for effect of plasma miR-101 expression on OS and RFS in Figure 1F and 1G.<br>Multivariate analysis for OS of gastric cancer patients in Table 3.
**Discussion**	
	Plasma miR-101 expression has prognostic value in patients underwent gastrectomy for gastric cancer. Study limited by sample size and by retrospective analysis.
